# Hemorrhagic risk of concomitant direct oral anticoagulants and fluoroquinolones: integration of pharmacovigilance and therapeutic drug monitoring

**DOI:** 10.3389/fphar.2026.1745035

**Published:** 2026-01-13

**Authors:** Jiao Xu, Yue-dong Li, Jun-ping Han, Chun-yan Duan, Feng-lun Zhao, Zi-yi Shang, Zhu Zhu, Zhan-hong Hu

**Affiliations:** Department of Pharmacy, The Second Affiliated Hospital of Soochow University, Suzhou, Jiangsu, China

**Keywords:** direct oral anticoagulants, drug-drug interactions, FAERS analysis, fluoroquinolones, therapeutic drug monitoring

## Abstract

**Background:**

Direct oral anticoagulants (DOACs) and fluoroquinolone antibiotics (FQNs) are often co-prescribed. A pharmacokinetic interaction is plausible, as DOACs are P-glycoprotein (P-gp) substrates and several FQNs can bind to and affect P-gp activity. However, robust clinical evidence characterizing the associated hemorrhagic reporting signal remains limited.

**Methods:**

We conducted an integrated pharmacoepidemiological and therapeutic drug monitoring (TDM) study. Disproportionality analysis was performed using FDA Adverse Event Reporting System (FAERS) data (2010–2025), calculating adjusted reporting odds ratios (adj. ROR) and Ω shrinkage values. Concurrently, a prospective observational cohort (n = 50) measured trough and peak plasma concentrations of dabigatran with and without levofloxacin or moxifloxacin.

**Results:**

FAERS analysis identified a significant reporting signal for bleeding for dabigatran coadministered with FQNs (adj. ROR = 4.68, 95% CI: 3.41–6.55), particularly levofloxacin (adj. ROR = 6.12) and ciprofloxacin (adj. ROR = 3.84). No significant signals were found for rivaroxaban, apixaban, or edoxaban. Consistently, TDM showed significantly elevated peak dabigatran concentrations with levofloxacin (133.36 ng/mL, *P* = 0.008) and moxifloxacin (138.20 ng/mL, *P* < 0.001) compared to monotherapy (65.34 ng/mL), alongside a numerical trend towards more bleeding events.

**Conclusion:**

This integrated analysis provides suggestive evidence for a pharmacokinetically plausible interaction that may increase the reporting odds of bleeding between dabigatran and certain FQNs (e.g., levofloxacin). Other DOACs appear safer with FQNs coadministration. For dabigatran patients requiring FQNs, alternative agents or enhanced monitoring should be considered.

## 1 Introduction

Direct oral anticoagulants (DOACs) are cornerstone therapies for stroke prevention in patients with non-valvular atrial fibrillation (NVAF) (Joglar et al., 2024; Ko et al., 2025) and for the treatment and prevention of venous thromboembolism (VTE) (Geerts et al., 2004). Compared with vitamin K antagonists (VKAs), DOACs exhibit a more favorable safety profile and fewer food and concomitant drug interactions. However, bleeding continues to represent a major clinical concern (Conway et al., 2017). Importantly, the four most commonly prescribed DOACs-dabigatran, rivaroxaban, apixaban, and edoxaban-are all substrates of the efflux transporter P-glycoprotein (P-gp) (Lund et al., 2017; Stangier et al., 2005). Notably, factor Xa inhibitors (particularly apixaban and rivaroxaban) additionally undergo CYP3A4-mediated metabolism to varying degrees (Mar et al., 2022). These pharmacokinetic differences may lead to heterogeneous changes in systemic exposure and bleeding outcomes when DOACs are co-administered with interacting medications (Mar et al., 2022; Wiggins et al., 2020).

Fluoroquinolones (FQNs), including ciprofloxacin, levofloxacin, and moxifloxacin, are broad-spectrum antimicrobial agents commonly prescribed for urinary tract, gastrointestinal, and respiratory infections (Chan and Bunce, 2017). Previous studies have indicated that certain FQNs can inhibit CYP3A4 activity (Zhou et al., 2024; Toda et al., 2009a; Toda et al., 2009b). In addition, evidence demonstrates that several FQNs binds to (Lin et al., 2025) and may even inhibit P-gp function (Sikri et al., 2004; de Lange et al., 2000), which may affect its transport function. This combined inhibition of key metabolic and efflux pathways poses a plausible pharmacological risk of increasing systemic exposure to concomitantly administered DOACs, which may consequently elevate the risk of bleeding. However, robust clinical evidence characterizing this drug-drug interaction (DDI) remains limited (Yagi et al., 2023).

Notably, the bleeding risk associated with DOAC-FQN coadministration may differ substantially among individual DOACs due to their distinct pharmacokinetic pathways and varying dependence on transporters and enzymes affected by FQNs. Dabigatran etexilate (DE) is one of the preferred oral anticoagulants for stroke prevention in patients with atrial fibrillation (AF) (Connolly et al., 2009). These patients often exhibit reduced cardiac output and pulmonary congestion due to chronic arrhythmia, which predisposes them to pulmonary infections during long-term anticoagulation therapy. Studies have indicated that the incidence of hospital-acquired pneumonia is approximately sevenfold higher in AF patients compared with those without AF (Zhu et al., 2015). Therefore, FQNs remain integral to the clinical management of infections in this patient population. Importantly, drug interaction-induced fluctuations in dabigatran plasma concentrations are a well-recognized risk factor for both bleeding and thrombotic events (Mar et al., 2022). Clinical studies have demonstrated that a doubling of dabigatran exposure can increase the risk of bleeding by 50%–300% (Pham et al., 2020). Nevertheless, the current prescribing information for DE, as with other DOACs, does not address potential interaction with FQNs, leaving the safety profile across different DOAC-FQN combinations insufficiently characterized.

Therefore, this study aimed to utilize the FDA Adverse Event Reporting System (FAERS) database to first assess the reporting signal for bleeding events associated with the concomitant use of different DOACs and fluoroquinolone agents, thereby elucidating the general trend of this potential interaction. Subsequently, based on clinical therapeutic drug monitoring (TDM) data, we focus specifically on DE to investigate the impact of its coadministration with commonly used FQNs (moxifloxacin and levofloxacin) on dabigatran plasma concentrations and clinical outcomes. The findings of this study are expected to provide preliminary evidence to inform clinical decision-making and enhance the safety of patients requiring combined anticoagulant and antimicrobial therapy.

## 2 Methods

### 2.1 FAERS database study

#### 2.1.1 Data source and extraction

Adverse event reports were extracted from the FAERS database (Subeesh et al., 2019) for the period from the first quarter (Q1) of 2010 to Q1 2025, covering the post-marketing surveillance periods of all DOACs and the specified FQNs. The dataset was curated by excluding duplicate entries, retaining only the most recent version of each case in accordance with FDA recommendations.

Reports were systematically categorized into three cohorts: (i) DOAC Monotherapy-reports listing a DOAC (dabigatran, rivaroxaban, apixaban, or edoxaban) as the “Primary Suspect (PS)” drug, with no FQN (levofloxacin, moxifloxacin, or ciprofloxacin) appearing in any other role (Secondary Suspect, Concomitant, or Interacting); (ii) FQN Monotherapy-reports listing an FQN as the PS drug, with no DOAC included in any other role; and (iii) Combination Therapy-reports listing either a DOAC as the PS drug and an FQN in another role, or *vice versa*.

Exclusion criteria comprised: (i) reports in which any of the study drugs were indicated for a bleeding-related disorder; and (ii) reports involving the concurrent use of more than one target DOAC or FQN within the same report. For pairwise DDI analysis, reports involving more than one DOAC or FQN were excluded to ensure that signals reflected specific drug pairs.

#### 2.1.2 Outcome definition and statistical analysis

Bleeding events were identified using a predefined set of Preferred Terms (PTs) from the Medical Dictionary for Regulatory Activities (MedDRA) (Supplementary Table S1 Annual distribution of bleeding events associated with DOACs reported in the FAERS database from 2010 to 2025.). Descriptive statistics were used to summarize demographic and clinical characteristics. Categorical variables were presented as frequencies and percentages and compared using the Chi-square or Fisher’s exact tests. Continuous variables were tested for normality with the Shapiro-Wilk test; data following a normal distribution were expressed as mean ± standard deviation (SD) and compared using one-way analysis of variance (ANOVA), whereas non-normally distributed data were reported as median (interquartile range, IQR) and compared using the Kruskal–Wallis test.

A multivariable logistic regression model was applied to calculate adjusted reporting odds ratios (adj. ROR) and 95% confidence intervals (CIs) for bleeding, adjusting for age, sex, body weight, reported diagnosis of NVAF, and FQNs use (Noguchi et al., 2019; Michel et al., 2017). The Ω shrinkage measure was employed as a dedicated statistical approach for DDI signal detection, with a signal considered significant when the lower bound of the 95% CI (Ω_0_._25_) exceeded zero (Noguchi et al., 2019; Noren et al., 2008).

All statistical analyses for FAERS data were performed using R software (version 4.4.0) and SPSS (version 27.0). A two-sided P-value <0.05 was considered statistically significant.

#### 2.1.3 Sensitivity analysis

Sensitivity analyses were conducted using amiodarone (a well-established P-gp/CYP3A4 inhibitor) (Ferri et al., 2022) as the positive control and atenolol (with no known pharmacokinetic interaction) as the negative control. To minimize potential reporting bias associated with the early post-marketing period of dabigatran, a subgroup analysis was performed using data restricted to the period from 2015 to 2025 (Kaba et al., 2014; Abou Kaoud et al., 2023). Additional analyses included the time to onset (TTO) of bleeding events and review by System Organ Class (SOC) categories.

### 2.2 Prospective cohort with therapeutic drug monitoring

#### 2.2.1 Study population and design

A prospective observational study was conducted in the Department of Cardiology at the Second Affiliated Hospital of Soochow University (Suzhou, China) between January 2018 and January 2022. The study protocol was approved by the hospital Ethics Committee (JD-LK-2018–069-01) and was conducted in accordance with the principles of the Declaration of Helsinki.

Adults diagnosed with NVAF who had been stably treated with dabigatran etexilate (110 mg twice daily) for at least 1 month were eligible for inclusion. Major exclusion criteria included: pregnancy; age <18 years; severe cardiac, hepatic, or renal dysfunction; active malignancy; history of renal transplantation or ongoing long-term dialysis; and concomitant use of known P-gp/CYP3A4 modulators (e.g., amiodarone, verapamil, clarithromycin, rifampin, or azole antifungals) (Steffel et al., 2018), antiplatelet agents, or other anticoagulants.

#### 2.2.2 Data collection and TDM

Baseline data were collected, including demographics, vital signs, medical history, concomitant medications, and laboratory parameters. Creatinine clearance (CrCl) was calculated using the Cockcroft-Gault equation. Stroke and bleeding risks were assessed using CHA_2_DS_2_-VASc and HAS-BLED scores, respectively.

Paired trough samples (collected immediately before the next dose) and peak samples (collected 2–3 h after dosing) were obtained from each participant. Plasma was separated by centrifugation and stored at −80 °C until analysis. Dabigatran plasma concentrations were quantified using a validated ultra-performance liquid chromatography-tandem mass spectrometry (UPLC-MS/MS) method, as previously described (Delavenne et al., 2012). Chromatographic separation was performed on a Hypersil GOLD C18 column under gradient elution conditions. Mass spectrometric detection was conducted in positive ionization mode, monitoring the mass transitions of m/z 472→m/z 289 for dabigatran and m/z 237→m/z 194 for the carbamazepine internal standard.

#### 2.2.3 Clinical outcomes and statistical analysis

Patients were followed for a period of 1 month after discharge. The composite endpoint of “any bleeding” included both major and minor bleeding events. All bleeding events were actively ascertained through structured patient interviews conducted at two predefined time points: prior to hospital discharge and 1 month after discharge. This follow-up window was selected to correspond to the typical duration of concomitant dabigatran–fluoroquinolone exposure in our cohort (generally 2–4 weeks) and to capture events occurring during and shortly after the co-exposure period. The most commonly observed bleeding presentations were gastrointestinal bleeding and urinary tract bleeding. Assessors of clinical outcomes were blinded to both patient group allocation and dabigatran plasma concentration results.

Bleeding events were recorded and categorized as either major or minor. Major bleeding was defined as a decrease in hemoglobin of ≥20 g/L, transfusion of ≥2 units of blood, or symptomatic bleeding involving a critical site or organ. All other bleeding episodes were classified as minor bleeding (Hori et al., 2013).

Statistical analysis for the cohort data was performed using SPSS software (version 27.0). Normality of continuous variables was assessed using the Shapiro-Wilk test. For normally distributed data, comparisons between two groups were made using Student’s t-test, and among more than two groups using ANOVA. For non-normally distributed data, the Mann-Whitney U test (two groups) or Kruskal–Wallis test (> two groups) was applied. Categorical variables were compared using Fisher’s exact test: an overall four-group comparison (DE only, DE + FQNs, DE + moxifloxacin, and DE + levofloxacin) was evaluated using the Fisher–Freeman–Halton exact test, and prespecified pairwise comparisons versus the DE-only group were performed using two-sided Fisher’s exact tests. A two-sided *P* value <0.05 was considered statistically significant.

Given the limited sample size and low event frequency in the TDM cohort, we conducted a *post hoc* power analysis for the binary endpoint “any bleeding”. Power was estimated for a two-sided comparison of two independent proportions (α = 0.05) using the observed bleeding rates in the combination arm (6/20) and the dabigatran-only arm (4/30). Group comparisons were evaluated using Fisher’s exact test; the power estimate was obtained using a standard normal approximation for two-proportion comparisons.

## 3 Results

### 3.1 FAERS database results

#### 3.1.1 Demographic and patient characteristics

**FIGURE 1:**
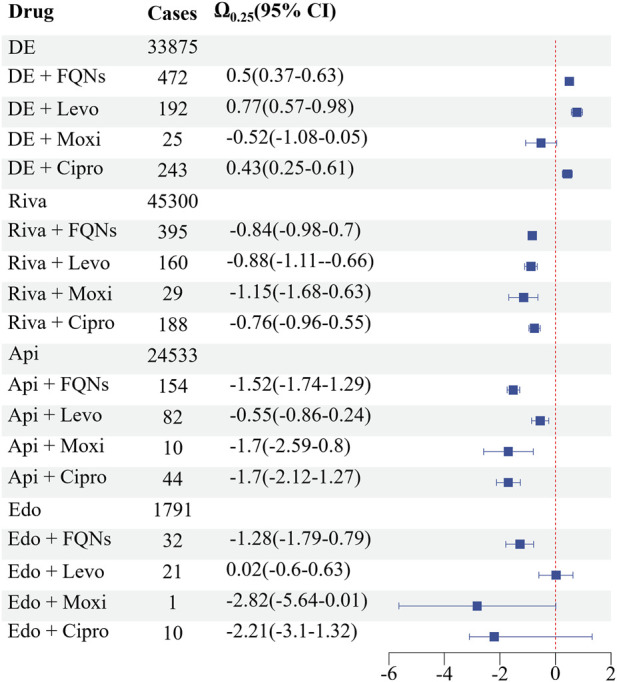
Demographic Characteristics From a total of 17,440,611 reports in the FAERS database, 253,645 were associated with DOACs and 76,710 with FQNs. Among these, 1,937 reports documented concomitant DOAC-FQN use, of which 759 (39.2%) involved bleeding events. When stratified by individual DOACs, dabigatran accounted for the highest number of bleeding reports under combination therapy (n = 320), followed by rivaroxaban (n = 265), apixaban (n = 142), and edoxaban (n = 23). *: For pairwise DDI analysis, reports involving concomitant use of multiple DOACs or FQNs were excluded to avoid confounding, resulting in slightly lower counts for individual drug pairs.

From a total of 17,440,611 reports in the FAERS database, 253,645 were associated with DOACs and 76,710 with FQNs. Among these, 1,937 reports documented concomitant DOAC-FQN use, of which 759 (39.2%) involved bleeding events. When stratified by individual DOACs (excluding reports involving more than one DOAC or FQN to ensure clean drug-pair signals), dabigatran accounted for the highest number of bleeding reports under combination therapy (n = 320), followed by rivaroxaban (n = 265), apixaban (n = 142), and edoxaban (n = 23). The sum of these individual DOAC-specific cases (n = 735) is slightly lower than the total number of bleeding events associated with any DOAC-FQN coadministration (n = 759) because the latter includes reports in which concomitant use of more than one DOAC or FQN was listed (Figure 1).

Dabigatran also exhibited the highest proportion of bleeding events among its total reports (45.1%), comparable to rivaroxaban (44.6%). Patients experiencing dabigatran-associated bleeding were the oldest (mean age: 74.8 ± 14.1 years). Notably, among the bleeding cases reported under combination therapy, those involving dabigatran demonstrated the highest case-fatality rate (29.7%) and the highest proportion of reports (74.8%) in which the DOAC–FQN combination was listed as suspect (Table 1). It should be emphasized that this reporter-assigned suspect coding in FAERS indicates association rather than causality.

**TABLE 1:** Characteristics and demographics of patients with bleeding adverse events associated with DOACs and fluoroquinolone use.

No. of total AEs	DE (57,763)	Riva (79,820)	Api (104,598)	Edo (3762)	*P* value
Total AEs					—
Bleeding	26,035 (45.1%)	35,593 (44.6%)	21,494 (20.5%)	1480 (39.3%)	​
Not bleeding	31,728 (54.9%)	44,227 (55.4%)	83,104 (79.4%)	2282 (60.7%)	​
Sex^*^					<0.001
Female	10,992 (42.2%)	16,179 (45.5%)	9001 (41.9%)	473 (32.0%)	​
Male	12,443 (47.8%)	14,526 (40.8%)	10,029 (46.7%)	604 (40.8%)	​
Missing	2600 (10.0%)	4888 (13.7%)	2464 (11.5%)	403 (27.2%)	​
Age^a^					<0.001
Age ±SD (Year)	74.8 ± 14.1	65.5 ± 18.6	72.1 ± 18.1	72.8 ± 21.3	​
Proportion of concomitant bleeding AE^b^					
FQNs	320 (74.8%, 320/428)	265 (38.7%, 265/685)	142 (20.2%, 142/702)	23 (26.7%, 23/86)	<0.001
Levo	136 (77.7%, 136/175)	107 (35.8%, 107/299)	73 (23.5%, 73/310)	14 (43.8%, 14/32)	<0.001
Moxi	20 (74.1%, 20/27)	25 (40.3%, 25/62)	9 (25%, 9/36)	1 (20%, 1/5)	<0.001
Cipro	157 (72.0%, 157/218)	118 (39.3%, 118/300)	50 (13.5%, 50/357)	8 (16.3%, 8/49)	<0.001
Proportion of concomitant death cases^c^					
FQNs	95 (29.7%, 95/320)	77 (29.1%, 77/265)	26 (18.3%, 26/142)	2 (8.7%, 2/23)	<0.01
Levo	52 (38.2%, 52/136)	46 (43.0%, 46/107)	9 (12.3%, 9/73)	1 (7.1%, 1/14)	<0.001
Moxi	2 (10.0%, 2/20)	6 (24.0%, 6/25)	8 (88.9%, 8/9)	1 (100%, 1/1)	<0.001
Cipro	39 (24.8%, 39/157)	16 (13.6%, 16/118)	9 (18.0%, 9/50)	—	—

*Levo,* levofloxacin; *Moxi,* moxifloxacin; *Riva* rivaroxaban.

Continuous variables were assessed for normality using the Shapiro-Wilk test. Normally distributed data were compared using one-way ANOVA; non-normally distributed data were analyzed using the Kruskal–Wallis test or Fisher test. A two-sided *P*-value <0.05 was considered statistically significant.

^a^ Data are presented only for cases in which bleeding occurred.

^b^ Proportion of bleeding events among all adverse events reported following concomitant therapy.

^c^ Proportion of fatal outcomes among reported bleeding events.

Temporal trend analysis revealed that dabigatran-related reports peaked during 2011–2014, while rivaroxaban-related reports were most frequent between 2015 and 2020 (Supplementary Figure S1).

#### 3.1.2 Bleeding-related DDI

Disproportionality analysis demonstrated a significantly elevated reporting odds for bleeding for dabigatran in combination with FQNs (adj.ROR = 4.68, 95% CI: 3.41–6.55). In contrast, no increased reporting odds were observed for rivaroxaban (adj.ROR = 0.54, 95% CI: 0.40–0.71), apixaban (adj.ROR = 0.32, 95% CI: 0.24–0.43), or edoxaban (adj.ROR = 0.10, 95% CI: 0.02–0.30). Among the FQNs, levofloxacin showed the strongest association with dabigatran-related bleeding (adj.ROR = 6.12, 95% CI: 3.66–11.01), followed by ciprofloxacin (adj.ROR = 3.84, 95% CI: 2.52–6.06). The point estimate for moxifloxacin was elevated but did not reach statistical significance (adj.ROR = 2.37, 95% CI: 0.76–8.86) (Table 2).

**TABLE 2:** Adjusted reporting odds ratios (adj.ROR) for bleeding events associated with DDI between DOACs and FQNs.

Drugs	adj.ROR (95% CI)	adj.ROR (95% CI) for DDI (FQNs)	adj.ROR (95% CI) for DDI (levo)	adj.ROR (95% CI) for DDI (moxi)	adj.ROR (95% CI) for DDI (cipro)
DE	1.54 (1.46–1.61)	4.68 (3.41–6.55)	6.12 (3.66–11.01)	2.37 (0.76–8.86)	3.84 (2.52–6.06)
Riva	1.23 (1.17–1.28)	0.54 (0.4–0.71)	0.34 (0.21–0.52)	0.92 (0.32–2.43)	0.86 (0.56–1.28)
Api	0.59 (0.57–0.62)	0.32 (0.24–0.43)	0.46 (0.32–0.67)	0 (0–0.06)	0.2 (0.12–0.32)
Edo	0.64 (0.56–0.74)	0.1 (0.02–0.3)	0.75 (0.09–6.29)	0.34 (0.02–2.35)	—

*Api,* apixaban; *Cipro,* ciprofloxacin; *DE,* dabigatran etexilate; *Edo* edoxaban; *FQNs*, fluoroquinolones; *Levo,* levofloxacin; *Moxi* moxifloxacin; *Riva* rivaroxaban.

**FIGURE 2:**
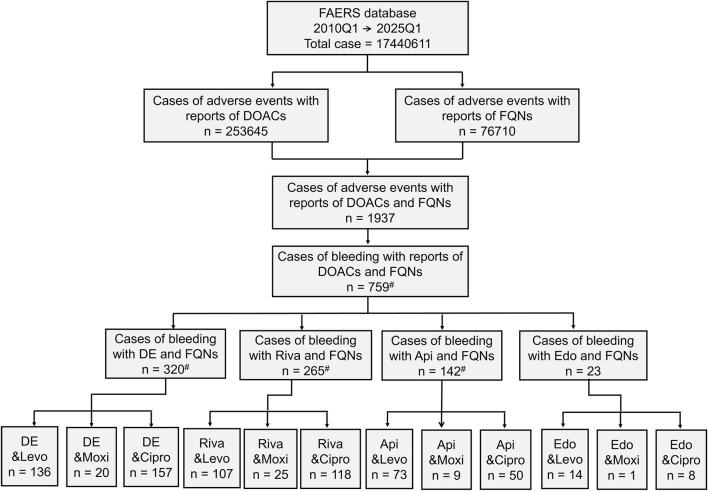
Bleeding-Related DDI The Ω shrinkage analysis further confirmed significant DDI signals for dabigatran coadministered with levofloxacin (Ω_0_._25_ = 0.77, 95% CI: 0.57–0.98) and ciprofloxacin (Ω_0_._25_ = 0.43, 95% CI: 0.25–0.61). No significant signals (Ω_0_._25_ < 0) were detected for any other DOAC-FQN combinations.

The Ω shrinkage analysis further confirmed significant DDI signals for dabigatran coadministered with levofloxacin (Ω_0_._25_ = 0.77, 95% CI: 0.57–0.98) and ciprofloxacin (Ω_0_._25_ = 0.43, 95% CI: 0.25–0.61). No significant signals (Ω_0_._25_ < 0) were detected for any other DOAC-FQN combinations (Figure 2).

Multivariable logistic regression analysis revealed that both rivaroxaban and dabigatran were associated with significantly higher odds of bleeding compared to apixaban and edoxaban. Increasing age was not significantly associated with bleeding risk, whereas male sex was modestly associated with higher odds of bleeding. Atrial fibrillation (AF) was identified as a strong independent predictor of bleeding events (Supplementary Table S2).

#### 3.1.3 Sensitivity and supplemental analyses

Sensitivity analyses confirmed the robustness of the findings and the validity of the analytical approach. A strong interaction signal was observed for the positive control pair, dabigatran-amiodarone (Ω_0_._25_ > 0), whereas no signal was detected for the negative control pair, dabigatran-atenolol (Ω_0_._25_ < 0). The significant signal for dabigatran-levofloxacin remained evident in the 2015–2025 subset analysis (adj.ROR = 59.6, 95% CI: 45.73–77.67, Ω_0_._25_ = 0.83) (Table 3).

**TABLE 3:** Sensitivity analysis of DDI signals for bleeding risk associated with DOACs and concomitant medications.

Drug	adj.ROR (95% CI) for DDI	Shrinkage analysis (Ω_0.25_)
DE-amiodarone	16.96 (16.14–17.82)	2.5E-05
DE-atenolol	16.12 (14.94–17.38)	−0.1
Publication date		
DE-levo (2015–2025)	59.6 (45.73–77.67)	0.83

*DE*, dabigatran etexilate; *Levo,* levofloxacin*; Riva,* rivaroxaban.

The TTO analysis indicated that most bleeding events-across both monotherapy and combination-therapy groups-occurred within the first 30 days of treatment (Supplementary Figure S2). SOC analysis revealed that concomitant DOAC-FQN use, particularly with dabigatran, amplified risk signals not only within the “Blood and Lymphatic System Disorders” but also within “Cardiac” and “Gastrointestinal” classes (Supplementary Figure S3).

### 3.2 Prospective TDM cohort findings

#### 3.2.1 Patient demographics and clinical outcomes

The prospective cohort consisted of 50 patients, including 30 receiving dabigatran monotherapy and 20 receiving combination therapy (12 with moxifloxacin and 8 with levofloxacin). The patient flow diagram for the TDM cohort is shown in Supplementary Figure S4. Baseline characteristics-including demographic variables, laboratory parameters, and CHA_2_DS_2_-VASc/HAS-BLED scores-were well balanced across the two groups (Supplementary Table S3).

The incidence of the composite endpoint of “any bleeding” was numerically higher in the FQN combination group (30.0%) than in the monotherapy group (13.3%), although this difference did not reach statistical significance (*P* > 0.05). Specifically, in the dabigatran-only group, there were 2 gastrointestinal (GI) bleeding events and 2 urinary tract bleeding events. In the dabigatran + levofloxacin group, there was 1 GI and 1 urinary bleeding event. In the dabigatran + moxifloxacin group, there was 1 GI and 3 urinary bleeding events. All bleeding events were minor and were identified as occult blood positivity on routine stool testing (fecal occult blood) and/or urinalysis (urine occult blood). No major bleeding events were observed during the follow-up period (Table 4).

**TABLE 4:** Comparison of clinical bleeding events among study groups.

Event	DE (30)	DE + FQNs (20)	DE + moxi (12)	DE + levo (8)	*P* ^a^ value
Major bleed	0	0	0	0	​
Any bleed	4 (13.33%)	6 (30%)	4 (33.3%)	2 (25%)	0.353
*P* ^b^ value	/	0.171	0.195	0.587	​

Data are presented as number of events (percentage).

*DE*, dabigatran etexilate; *FQNs*, fluoroquinolones; *Levo* levofloxacin, *Moxi* moxifloxacin.

Statistical note.

Categorical outcomes (including bleeding events) were compared using two-sided exact tests.

^a^ Overall four-group comparison (DE, only, DE + FQNs, DE + Moxi, and DE + Levo) was evaluated using the Fisher–Freeman–Halton exact test.

^b^ Prespecified pairwise comparisons versus the DE-only group were performed using two-sided Fisher’s exact tests.

A two-sided *P* value <0.05 was considered statistically significant.

#### 3.2.2 Dabigatran plasma concentrations

**FIGURE 3:**
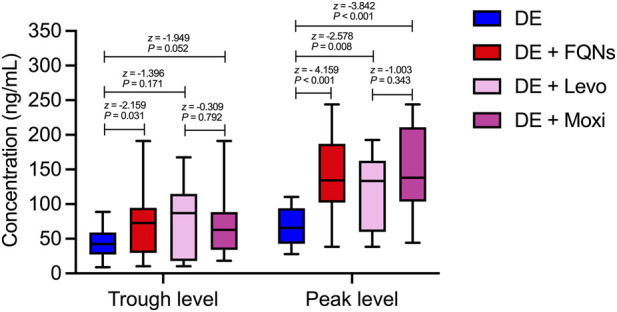
Dabigatran Plasma Concentrations Both trough and peak plasma concentrations of dabigatran were significantly higher in the combination group compared with the monotherapy group (Trough: 72.71 vs. 65.34 ng/mL, *P* = 0.031, Peak: 134.36 vs. 65.34 ng/mL, *P* < 0.001). Subgroup analysis further confirmed that peak diabigatran concentrations were significantly increased during coadministration with levofloxacin (133.36 ng/mL, *P* = 0.008) and moxifloxacin (138.20 ng/mL, *P* < 0.001). No significant differences were observed in trough concentrations among subgroups, nor between the two fluoroquinolone groups in either peak or trough values (all *P* > 0.05).

The TDM data provided mechanistic evidence supporting the pharmacovigilance findings. Both trough and peak plasma concentrations of dabigatran were significantly higher in the combination group compared with the monotherapy group (Trough: 72.71 vs. 42.26 ng/mL, *P* = 0.031, Peak: 134.36 vs. 65.34 ng/mL, *P* < 0.001). Subgroup analysis further confirmed that peak diabigatran concentrations were significantly increased during coadministration with levofloxacin (133.36 ng/mL, *P* = 0.008) and moxifloxacin (138.20 ng/mL, *P* < 0.001). No significant differences were observed in trough concentrations among subgroups, nor between the two fluoroquinolone groups in either peak or trough values (all *P* > 0.05) (Figure 3).

## 4 Discussion

This real-world study integrated pharmacovigilance data from the FAERS database with TDM and clinical outcomes to evaluate the potential reporting signals for bleeding associated with the concomitant use of DOACs and FQNs. Our findings indicate that the coadministration of dabigatran with levofloxacin or ciprofloxacin is associated with a significantly increased reporting odds of hemorrhage, supported by both disproportionality analysis and in the case of levofloxacin-elevated dabigatran plasma concentrations. In contrast, other DOACs (rivaroxaban, apixaban, and edoxaban) did not exhibit significant interaction signals with FQNs in the FAERS analysis, suggesting that they may represent safer alternatives when combination therapy is required.

The FAERS database, despite its inherent limitations such as underreporting and potential confounding, remains a valuable resource for detecting signals of rare but clinically meaningful drug-drug interactions (DDIs) (Noguchi et al., 2019; Noguchi et al., 2021). Our use of both adjusted reporting odds ratios (adj.ROR) and the Ω shrinkage measure-a robust statistical method for identifying DDIs in spontaneous reporting systems-enhances the reliability of our findings. The significant signals observed for dabigatran-levofloxacin (adj.ROR = 6.12, Ω_0_._25_ > 0) and dabigatran-ciprofloxacin (adj.ROR = 3.84, Ω_0_._25_ > 0) are mechanistically plausible, given the known inhibitory and binding effects of these FQNs onP-gp. It is noteworthy that our prospective TDM cohort did not include patients receiving dabigatran-ciprofloxacin coadministration. This limitation resulted from the absence of blood samples from such patients, as ciprofloxacin was not routinely stocked or prescribed on the cardiology ward during the study period, preventing the collection of pharmacokinetic data. Therefore, the FAERS signal for dabigatran–ciprofloxacin is supported solely by pharmacovigilance evidence and known pharmacological mechanisms, but not by direct concentration measurements in our study.

Our FAERS results are further validated by sensitivity analyses. The positive control (amiodarone)- a known P-gp and CYP3A4 inhibitor (Ferri et al., 2022) -showed a significant interaction signal with dabigatran (adj.ROR = 16.96, Ω_0_._25_ > 0), whereas the negative control (atenolol) demonstrated no signal (Ω_0_._25_ < 0). Moreover, to address potential litigation-related reporting bias during the period 2010–2014 when a trial was ongoing against Boehringer Ingelheim (the manufacturer of dabigatran) (Charlton and Redberg, 2014), we re-analyzed data from 2015 to 2025. Such litigation could have stimulated heightened adverse event reporting irrespective of the true pharmacological risk, potentially inflating signal estimates in the earlier years. This sensitivity analysis, conducted to mitigate such positive reporting bias, revealed that the dabigatran–levofloxacin signal remained significant. The persistence of this signal after excluding the potentially biased earlier period, further strengthens the credibility of our results. The agreement across analytical methods, the consistency with pharmacological mechanisms, and the confirmatory TDM data for levofloxacin provide multi-dimensional support for the potential clinical relevance of this interaction.

Our TTO and SOC analyses offer additional clinical insights. In the DOAC-FQN combined therapy, after aligning the duration of DOAC with FQN, the TTO analysis revealed that, bleeding events with combined medication therapy almost all occur within the first 30 days. Based on this time characteristic, it is recommended that the initial stage (the first 30 days) of the combination of the two drugs should be set as a key monitoring window for bleeding risk in clinical practice. The SOC analysis demonstrated that DOAC–FQN coadministration, especially involving dabigatran, not only amplified signals for “Blood and Lymphatic System Disorders” but also increased reporting frequencies in “Cardiac” and “Gastrointestinal” categories. This suggests that enhanced anticoagulant exposure may have broader systemic consequences, manifesting as gastrointestinal discomfort or cardiac arrhythmias, which could serve as early indicators of drug-related toxicity. It should be noted, however, these signals may also reflect manifestations of underlying acute illness rather than solely DDI effects.

In our clinical cohort, a numerical increase in any bleeding event was observed with moxifloxacin coadministration (33.3% vs. 13.33% in controls), paralleling the trend seen with levofloxacin (25.00%). However, these differences did not reach statistical significance, and no major bleeding events occurred during follow-up—likely due to the limited sample size. Furthermore, TDM results revealed no significant differences in trough or peak dabigatran concentrations between the moxifloxacin and levofloxacin subgroups. Notably, although TDM confirmed significantly elevated peak dabigatran concentrations during coadministration with moxifloxacin, this interaction did not produce a significant FAERS signal (adj.ROR = 2.37, 95% CI: 0.76 = 8.86, Ω_0_._25_ < 0). Given the discrepancy between pharmacokinetic and pharmacovigilance signals, along with the limited sample size and lack of statistical significance in clinical outcomes, the available evidence remains inconclusive regarding the bleeding risk associated with dabigatran–moxifloxacin coadministration. In clinical practice, prescribers may consider this uncertainty when choosing antimicrobial therapy for patients on dabigatran, particularly in high-risk settings, and may opt for alternative agents or enhanced monitoring where appropriate.

The marked difference in interaction signals between dabigatran and other DOACs (rivaroxaban, apixaban, and edoxaban) when combined with FQNs can be explained by their distinct pharmacokinetic pathways and dependence on P-gp and CYP3A4. FQNs were reported to bind to p-gp using their typical 4-quinolone parent nucleus structure (Lin et al., 2025), and studies also have confirmed that levofloxacin and ciprofloxacin can inhibit P-gp to some degree (Sikri et al., 2004; de Lange et al., 2000); however, their CYP3A4 inhibition is weak and variable. Dabigatran relies primarily on P-gp-mediated efflux for clearance (Lund et al., 2017), with minimal CYP involvement (Stangier et al., 2005), rendering it particularly susceptible to potent P-gp inhibition or functional modulation by agents such as levofloxacin and ciprofloxacin. This leads to enhanced absorption, reduced elimination, and consequently, increased plasma exposure and bleeding risk. In contrast, rivaroxaban and apixaban are dual substrates of both P-gp and CYP3A4 (Mar et al., 2022). This pharmacokinetic duality allows for functional compensation when one pathway is impaired. An analogous scenario is illustrated in the study of the anticancer drug vinorelbine (Lagas et al., 2012), which is also a dual substrate of P-gp and CYP3A. In that model, genetic knockout of Cyp3a resulted in compensatory upregulation of alternative metabolic pathways—notably carboxylesterase-mediated conversion to an active metabolite—therein maintaining overall drug clearance despite loss of the primary CYP3A route. Similarly, for factor-Xa inhibitors such as rivaroxaban and apixaban, when P-gp is occupied or inhibited by FQNs, CYP3A4 activity may be upregulated or its catalytic efficiency enhanced, thereby compensating for the reduced transporter-mediated clearance and limiting a clinically meaningful increase in systemic exposure. However, this remains a speculative extrapolation based on analogous pharmacological models, and further mechanistic investigations are warranted to substantiate this hypothesis, which represents an important direction for future research.

Although an online post-marketing signal aggregation platform (eHealthMe) reports a potential association between dabigatran (Pradaxa) and bleeding events in the setting of concomitant fluoroquinolone (FQN) use, this information is derived from spontaneous reports and should be interpreted as hypothesis-generating rather than confirmatory (eHealthMe, 2025).

Consequently, DDIs between DOACs and FNQs are not explicitly addressed in current product labeling or clinical guidelines (Joglar et al., 2024). A recent real-world study reported that the bleeding risk associated with DOACs-FNQs combinations, compared with DOAC-doxycycline, was modest (Yagi et al., 2023). However, dabigatran accounted for only 10.6% of DOACs in that cohort, likely diluting any dabigatran-specific signal, and no subgroup analysis by individual DOAC was performed. Our study therefore provides complementary evidence, integrating pharmacovigilance data, TDM findings, and clinical outcomes, confirming that dabigatran carries a higher bleeding risk when coadministered with FQNs compared with other DOACs.

Several limitations warrant acknowledgment. First, the FAERS database is subject to underreporting, reporting bias, incomplete data entry and the potential for exposure misclassification, etc. Regarding exposure misclassification, although reports related to PS drugs were used for analysis, it is not possible to rule out the inclusion of temporally unrelated or previously discontinued medications. The database also entails several inherent constraints, including: Uncontrolled confounding: Important clinical variables—such as genetic polymorphisms in drug transporters, variations in renal function, and concomitant use of other medications (e.g., antiplatelet agents or proton pump inhibitors) —are not systematically captured, precluding their adjustment in our analysis. Although PS -based analyses, head-to-head comparisons, along with positive/negative controls and sensitivity analyses, were used to partially mitigate these biases, the findings derived from FAERS should be interpreted as signaling associative relationships rather than establishing causality. Prospective studies with richer clinical data are needed to confirm these signals. Second, the prospective TDM cohort was relatively small and bleeding events were infrequent, with no major bleeding observed. The *post hoc* power for detecting between-group differences in “any bleeding” was limited (∼30%), and therefore outcome comparisons should be interpreted as exploratory/hypothesis-generating rather than definitive. Larger, adequately powered prospective outcome studies are warranted. Finally, the discrepancy between the pharmacokinetic effect of moxifloxacin and its FAERS signal underscores the challenge of interpreting DDI signals from spontaneous reports alone and highlights a key limitation of our integrated approach. This divergence illustrates that increases in drug exposure do not invariably translate into detectable safety signals in spontaneous reporting systems, and cautions against assuming a direct causative relationship between plasma concentration elevations and clinical bleeding risk for all interacting pairs.

## 5 Conclusion

In conclusion, this study provides robust evidence that the coadministration of dabigatran with levofloxacin or ciprofloxacin is associated with increased dabigatran plasma exposure and a heightened risk of bleeding. Clinicians should exercise vigilance when prescribing these combinations and consider alternative antimicrobial or anticoagulants whenever feasible. Future prospective studies involving larger, well-characterized cohorts are warranted to validate these findings and to inform individualized management strategies for patients requiring combined anticoagulant and antimicrobial therapy.

## Data Availability

The original contributions presented in the study are included in the article/Supplementary Material, further inquiries can be directed to the corresponding authors.
