# Supplementary material for: Hemorrhagic risk of concomitant direct oral anticoagulants and fluoroquinolones: integration of pharmacovigilance and therapeutic drug monitoring
Source: Front Pharmacol. 2026 Jan 13;17:1745035. doi: 10.3389/fphar.2026.1745035 (PMC12835336; doi:10.3389/fphar.2026.1745035)
Supplement: Supplementary file 1 [file DataSheet1.pdf]

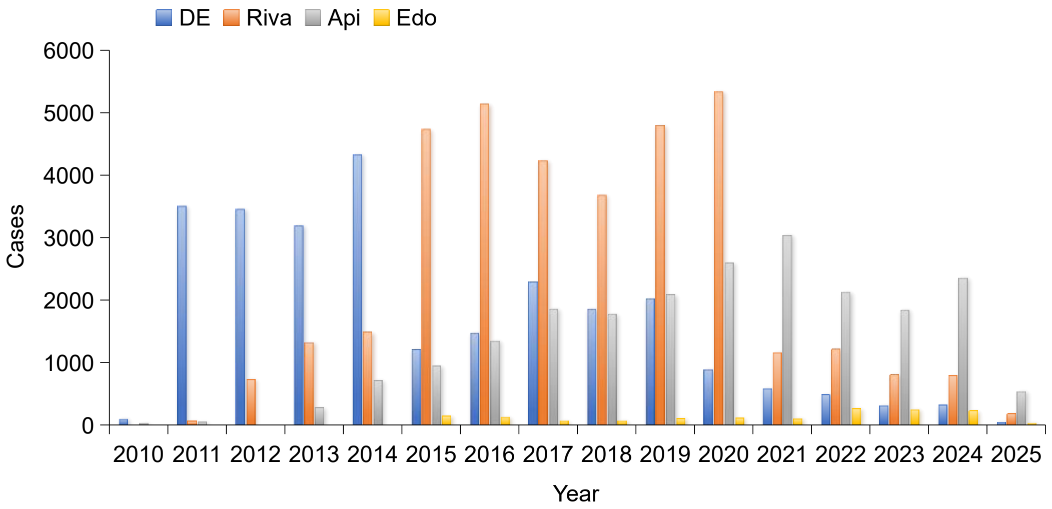

Figure S1 Annual distribution of bleeding events associated with direct oral anticoagulants (DOACs) reported in the FAERS database from 2010 to 2025.
Api apixaban, *DE* dabigatran etexilate, *Edo* edoxaban, *Riva* rivaroxaban

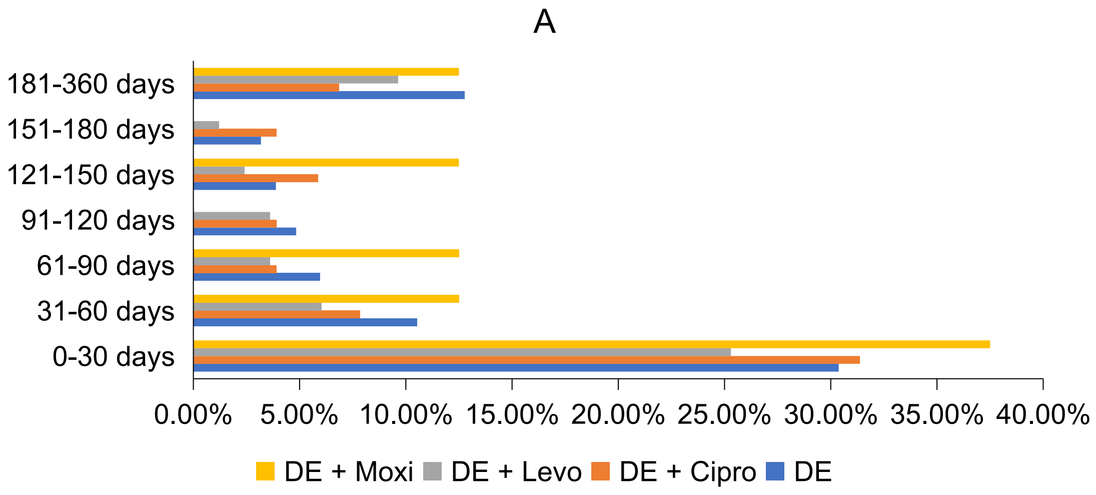

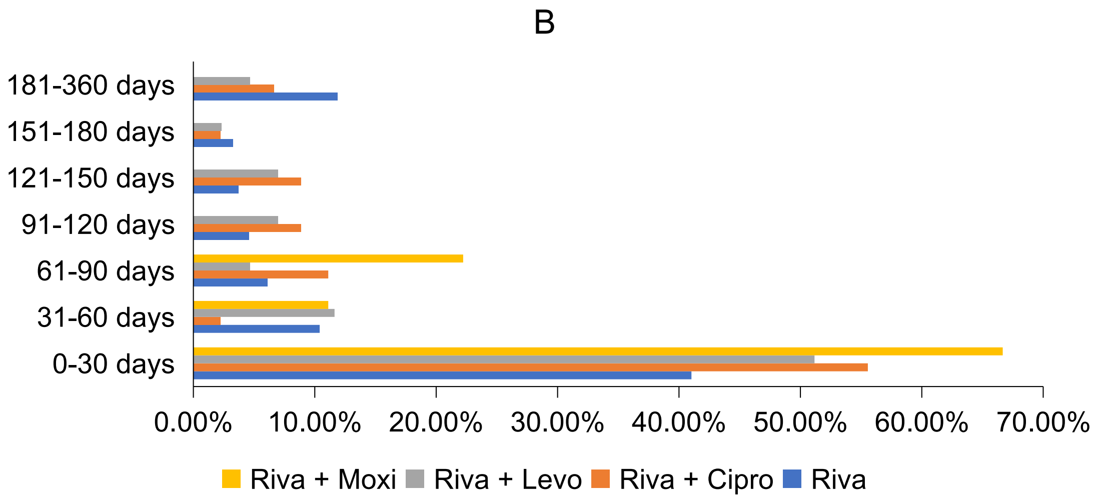
B
181-360 days
151-180 days
121-150 days
91-120 days
61-90 days
31-60 days
0-30 days
0.00% 10.00% 20.00% 30.00% 40.00% 50.00% 60.00% 70.00%
Riva + Moxi
Riva + Levo
Riva + Cipro
Riva

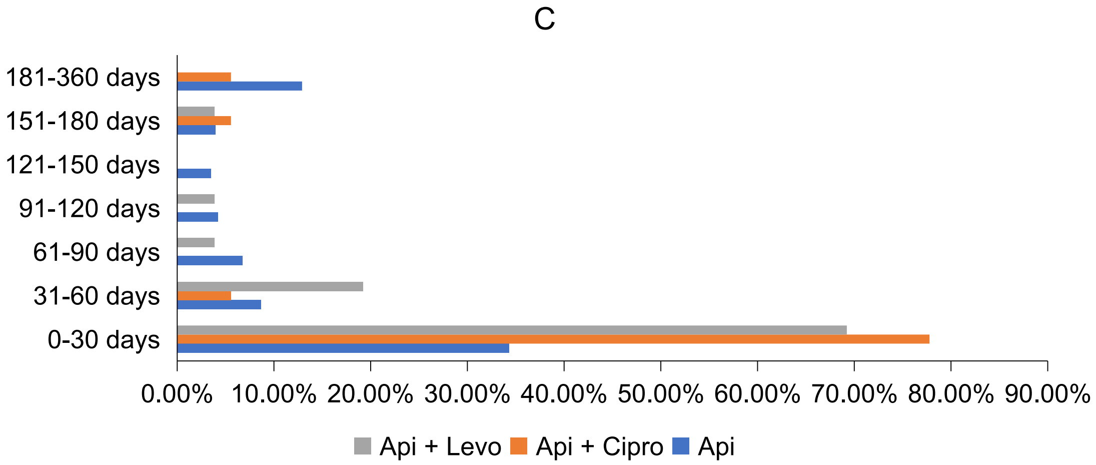
C
181-360 days
151-180 days
121-150 days
91-120 days
61-90 days
31-60 days
0-30 days
0.00% 10.00% 20.00% 30.00% 40.00% 50.00% 60.00% 70.00% 80.00% 90.00%
Api + Levo
Api + Cipro
Api

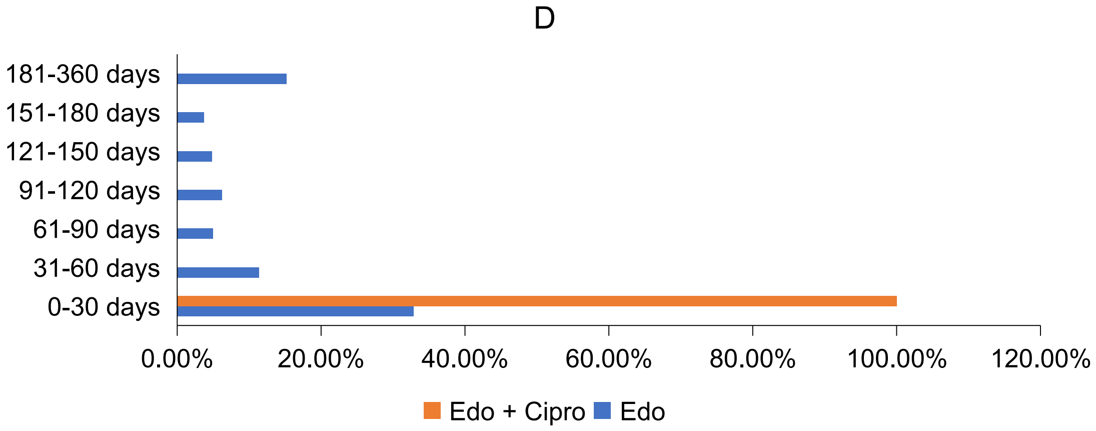
D
181-360 days
151-180 days
121-150 days
91-120 days
61-90 days
31-60 days
0-30 days
0.00% 20.00% 40.00% 60.00% 80.00% 100.00% 120.00%
Edo + Cipro
Edo

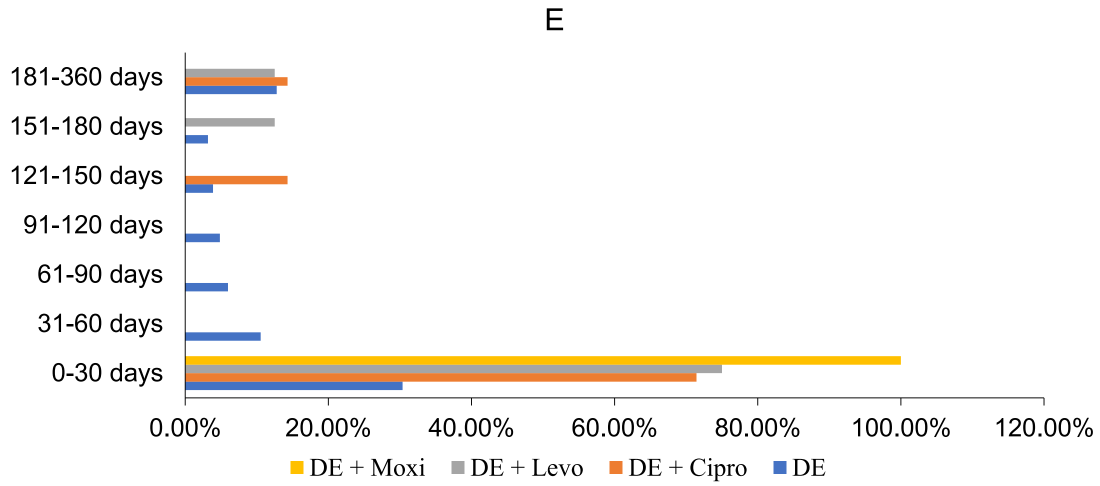
E
181-360 days
151-180 days
121-150 days
91-120 days
61-90 days
31-60 days
0-30 days
0.00%
20.00%
40.00%
60.00%
80.00%
100.00%
120.00%
DE + Moxi
DE + Levo
DE + Cipro
DE

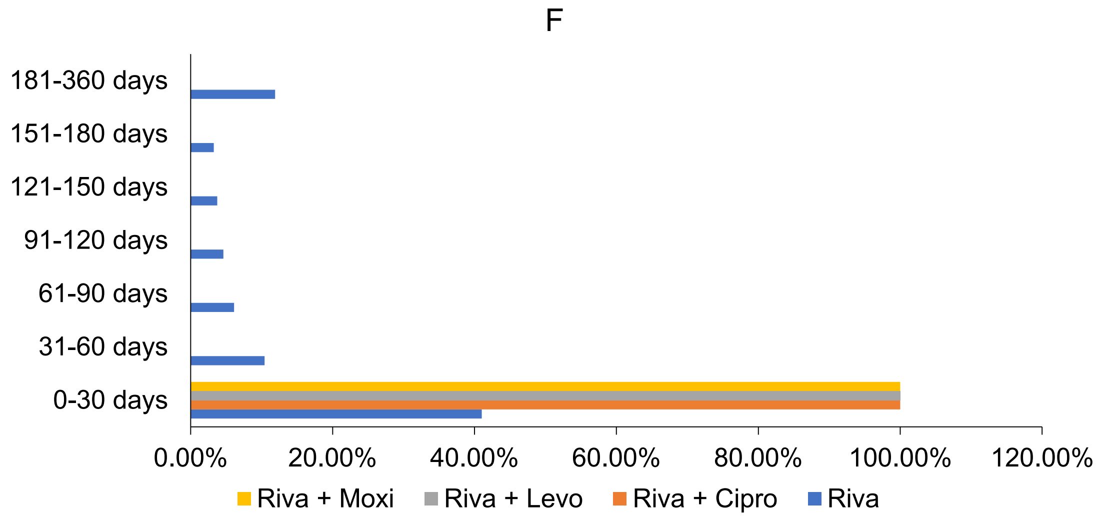
F
181-360 days
151-180 days
121-150 days
91-120 days
61-90 days
31-60 days
0-30 days
0.00%
20.00%
40.00%
60.00%
80.00%
100.00%
120.00%
Riva + Moxi
Riva + Levo
Riva + Cipro
Riva

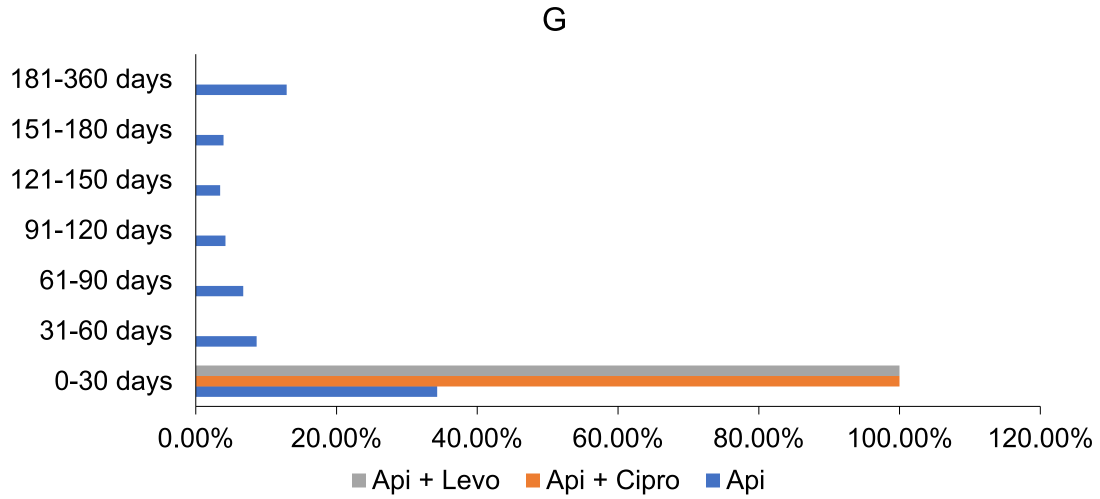
G
181-360 days
151-180 days
121-150 days
91-120 days
61-90 days
31-60 days
0-30 days
0.00%
20.00%
40.00%
60.00%
80.00%
100.00%
120.00%
Api + Levo
Api + Cipro
Api

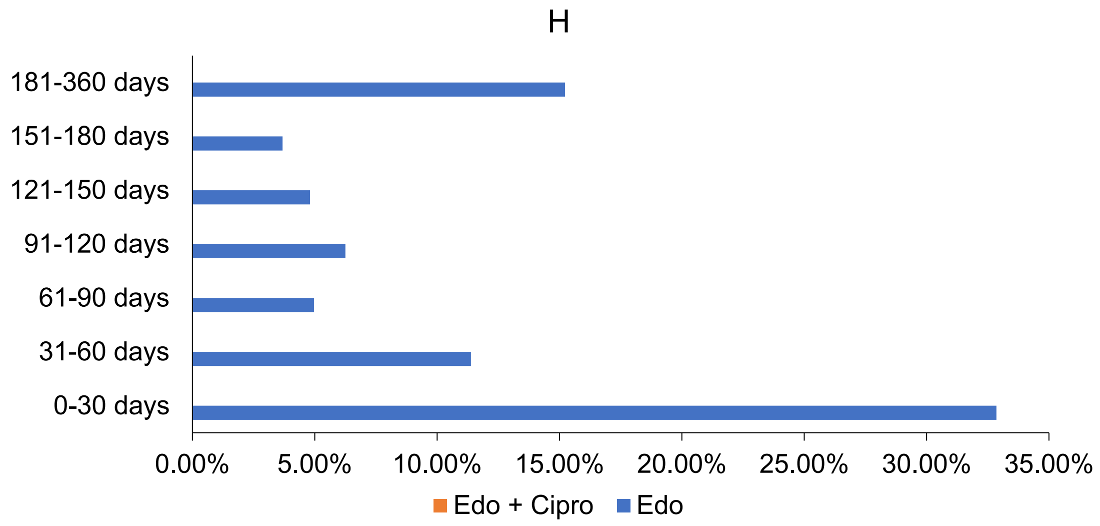

Figure S2 Distribution of time-to-onset (TTO) for bleeding events. The histogram illustrates the time to onset of bleeding events, categorized into 30-day intervals. Panels A-D depict the TTO distributions for dabigatran etexilate, rivaroxaban, apixaban, and edoxaban, respectively, under both monotherapy and combination therapy conditions. Panels E-H depict the TTO distributions for dabigatran etexilate, rivaroxaban, apixaban, and edoxaban respectively after data alignment with standard FQN duration.

Api apixaban, *Cipro* ciprofloxacin, *DE* dabigatran etexilate, *Edo* edoxaban, *Levo* levofloxacin, *Moxi* moxifloxacin, *Riva* rivaroxaban

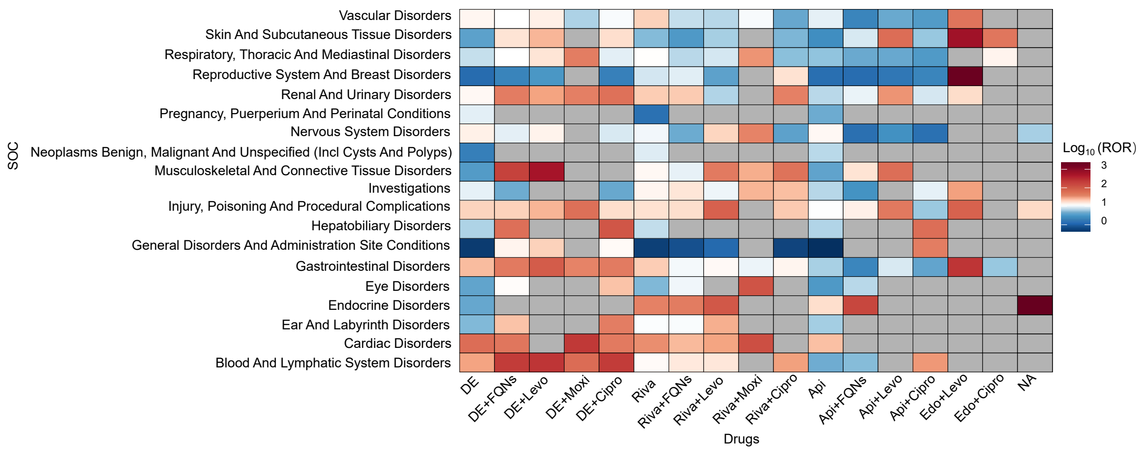

Figure S3 Heatmap for the direct oral anticoagulants and fluoroquinolones interaction. *Api* apixaban, *Cipro* ciprofloxacin, *DE* dabigatran etexilate, *Edo* edoxaban, *FQNs* fluoroquinolones, *Levo* levofloxacin, *Moxi* moxifloxacin, *Riva* rivaroxaban

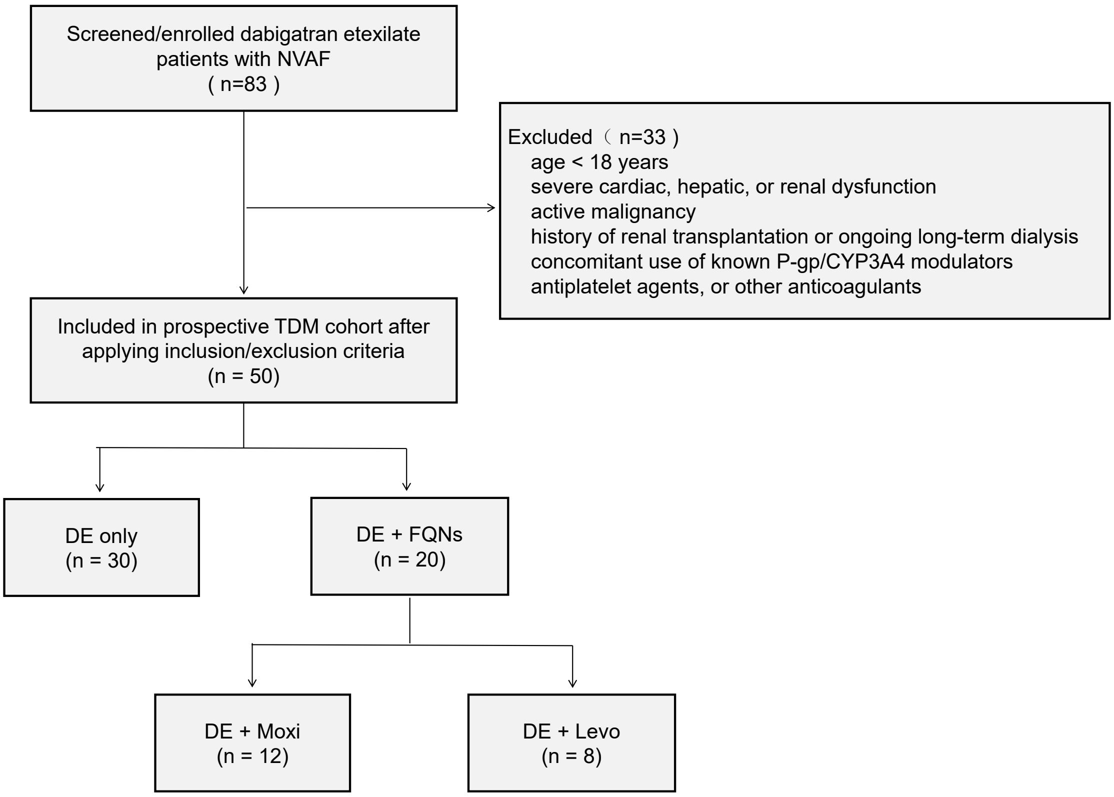

Figure S4 Patient flow dabigatran for the prospective TDM cohort.
FQNs fluoroquinolones, *Levo* levofloxacin, *Moxi* moxifloxacin, *DE* dabigatran etexilate

Table S1 A full list of preferred terms (PTs) for bleeding-related adverse events.

No.	PTs	No.	PTs
1	GASTRIC HAEMORRHAGE	192	HAEMORRHAGE NEONATAL
2	GASTROINTESTINAL HAEMORRHAGE	193	SKIN NEOPLASM BLEEDING
3	HAEMATURIA	194	HAEMATOTYMPANUM
4	HAEMATEMESIS	195	PURPURA FULMINANS
5	HAEMORRHAGE	196	PELVIC HAEMORRHAGE
6	HAEMOPTYSIS	197	PURPURA NON-THROMBOCYTOPENIC
7	WOUND HAEMORRHAGE	198	CEPHALHAEMATOMA
8	CEREBROVASCULAR ACCIDENT	199	UTERINE HAEMATOMA
9	HAEMATOCHEZIA	200	SCROTAL HAEMATOCOELE
10	HAEMARTHROSIS	201	SPINAL EPIDURAL HAEMORRHAGE
11	SUBARACHNOID HAEMORRHAGE	202	ADRENAL HAEMATOMA
12	VAGINAL HAEMORRHAGE	203	COITAL BLEEDING
13	SUBDURAL HAEMATOMA	204	FOETAL-MATERNAL HAEMORRHAGE
14	RECTAL HAEMORRHAGE	205	POLYMENORRHAGIA
15	ECCHYMOSIS	206	UMBILICAL HAEMORRHAGE
16	LOWER GASTROINTESTINAL HAEMORRHAGE	207	BREAST HAEMATOMA
17	TUMOUR HAEMORRHAGE	208	TOOTH SOCKET HAEMORRHAGE

18	EXSANGUINATION	209	INJECTION SITE HAEMATOMA
19	HAEMATOMA	210	OVARIAN HAEMORRHAGE
20	MELAENA	211	BRONCHIAL HAEMORRHAGE
21	UPPER GASTROINTESTINAL HAEMORRHAGE	212	RESPIRATORY TRACT HAEMORRHAGE NEONATAL
22	OESOPHAGEAL VARICES HAEMORRHAGE	213	SPONTANEOUS HAEMATOMA
23	CEREBRAL HAEMORRHAGE	214	VENOUS HAEMORRHAGE
24	CONJUNCTIVAL HAEMORRHAGE	215	HAEMORRHAGIC DISEASE OF NEWBORN
25	INCREASED TENDENCY TO BRUISE	216	INFUSION SITE HAEMATOMA
26	MUSCLE HAEMORRHAGE	217	BROAD LIGAMENT HAEMATOMA
27	RETROPERITONEAL HAEMORRHAGE	218	PERINEAL HAEMATOMA
28	EYE HAEMORRHAGE	219	CILIARY BODY HAEMORRHAGE
29	INJECTION SITE HAEMORRHAGE	220	URETERIC HAEMORRHAGE
30	MOUTH HAEMORRHAGE	221	LIP HAEMATOMA
31	HAEMORRHAGIC STROKE	222	BASAL GANGLIA HAEMORRHAGE
32	EPISTAXIS	223	LARYNGEAL HAEMORRHAGE
33	HAEMORRHAGE INTRACRANIAL	224	ARTERIOVENOUS FISTULA SITE HAEMATOMA
34	VITREOUS HAEMORRHAGE	225	HAEMORRHAGIC URTICARIA
35	CEREBELLAR HAEMORRHAGE	226	INTRA-ABDOMINAL HAEMATOMA
36	MUCOSAL HAEMORRHAGE	227	VULVAL HAEMATOMA
37	BLOOD URINE PRESENT	228	PELVIC HAEMATOMA OBSTETRIC
38	HAEMORRHAGIC DIATHESIS	229	NAEVUS HAEMORRHAGE
39	ENTEROCOLITIS HAEMORRHAGIC	230	ABDOMINAL WALL HAEMATOMA
40	PURPURA	231	BLOODY PERITONEAL EFFLUENT
41	DIARRHOEA HAEMORRHAGIC	232	ABDOMINAL WALL HAEMORRHAGE
42	PULMONARY HAEMORRHAGE	233	HAEMORRHAGIC EROSIVE GASTRITIS
43	GASTRIC ULCER HAEMORRHAGE	234	PHARYNGEAL HAEMATOMA
44	ANASTOMOTIC HAEMORRHAGE	235	THYROID HAEMORRHAGE
45	PULMONARY ALVEOLAR HAEMORRHAGE	236	CERVIX HAEMATOMA UTERINE
46	HAEMORRHOIDAL HAEMORRHAGE	237	AURICULAR HAEMATOMA
47	GINGIVAL BLEEDING	238	BRAIN STEM STROKE
48	CEREBRAL HAEMORRHAGE NEONATAL	239	INTRAPARTUM HAEMORRHAGE
49	HENOCH-SCHONLEIN PURPURA	240	AORTIC INTRAMURAL HAEMATOMA
50	DIVERTICULUM INTESTINAL HAEMORRHAGIC	241	SUBGALEAL HAEMATOMA
51	HEPATIC HAEMATOMA	242	ANAL ULCER HAEMORRHAGE
52	EXTRADURAL HAEMATOMA	243	PURPURA NEONATAL

53	DUODENAL ULCER HAEMORRHAGE	244	INTESTINAL HAEMATOMA
54	PETECHIAE	245	GASTRIC OCCULT BLOOD POSITIVE
55	RETINAL HAEMORRHAGE	246	PARANASAL SINUS HAEMATOMA
56	POSTPARTUM HAEMORRHAGE	247	CEREBRAL MICROHAEMORRHAGE
57	HEPATIC HAEMORRHAGE	248	RENAL CYST HAEMORRHAGE
58	INCISION SITE HAEMORRHAGE	249	UMBILICAL CORD HAEMORRHAGE
59	MELAENA NEONATAL	250	PENILE HAEMATOMA
60	PITUITARY HAEMORRHAGE	251	CHOROIDAL HAEMATOMA
61	OESOPHAGITIS HAEMORRHAGIC	252	SUBCHORIONIC HAEMORRHAGE
62	ULCER HAEMORRHAGE	253	BASAL GANGLIA STROKE
63	HAEMOTHORAX	254	LACRIMAL HAEMORRHAGE
64	SMALL INTESTINAL HAEMORRHAGE	255	LARYNGEAL HAEMATOMA
65	INTRA-ABDOMINAL HAEMORRHAGE	256	MESENTERIC HAEMATOMA
66	VASCULAR PURPURA	257	BRAIN STEM MICROHAEMORRHAGE
67	CEREBRAL HAEMATOMA	258	HAEMORRHAGE IN PREGNANCY
68	HAEMORRHAGIC TRANSFORMATION STROKE	259	MESENTERIC HAEMORRHAGE
69	MENOMETRORRHAGIA	260	UMBILICAL HAEMATOMA
70	UTERINE HAEMORRHAGE	261	PERIORBITAL HAEMORRHAGE
71	HAEMORRHAGE SUBCUTANEOUS	262	WOUND HAEMATOMA
72	HAEMORRHAGE URINARY TRACT	263	CENTRAL NERVOUS SYSTEM HAEMORRHAGE
73	HAEMORRHAGIC CEREBRAL INFARCTION	264	SUBRETINAL HAEMATOMA
74	INTESTINAL HAEMORRHAGE	265	INTRAOCULAR HAEMATOMA
75	URINARY BLADDER HAEMORRHAGE	266	CHRONIC PIGMENTED PURPURA
76	GASTRITIS HAEMORRHAGIC	267	SUBCHORIONIC HAEMATOMA
77	BREAST HAEMORRHAGE	268	SPINAL EPIDURAL HAEMATOMA
78	GENITAL HAEMORRHAGE	269	SPINAL SUBDURAL HAEMATOMA
79	BLOOD BLISTER	270	VITREOUS HAEMATOMA
80	RETROPERITONEAL HAEMATOMA	271	TOOTH PULP HAEMORRHAGE
81	HAEMORRHAGIC OVARIAN CYST	272	PERIPARTUM HAEMORRHAGE
82	CYSTITIS HAEMORRHAGIC	273	BRAIN STEM HAEMATOMA
83	INTRAVENTRICULAR HAEMORRHAGE	274	GASTROINTESTINAL POLYP HAEMORRHAGE
84	HAEMATOSPERMIA	275	STOMA SITE HAEMORRHAGE
85	PERICARDIAL HAEMORRHAGE	276	TRAUMATIC HAEMOTHORAX
86	EAR HAEMORRHAGE	277	SPONTANEOUS HAEMORRHAGE
87	PANCREATITIS HAEMORRHAGIC	278	CEREBELLAR MICROHAEMORRHAGE
88	SUBDURAL HAEMORRHAGE	279	PAROTID GLAND HAEMORRHAGE
89	PHARYNGEAL HAEMORRHAGE	280	LYMPH NODE HAEMORRHAGE
90	GASTROINTESTINAL ULCER HAEMORRHAGE	281	EPIDURAL HAEMORRHAGE

91	OESOPHAGEAL HAEMORRHAGE	282	NEONATAL GASTROINTESTINAL HAEMORRHAGE
92	ARTERIAL HAEMORRHAGE	283	EYELID HAEMATOMA
93	SHOCK HAEMORRHAGIC	284	DEEP DISSECTING HAEMATOMA
94	THALAMUS HAEMORRHAGE	285	INTERNAL HAEMORRHAGE
95	HAEMORRHAGIC DISORDER	286	PERINATAL STROKE
96	RENAL HAEMORRHAGE	287	SPINAL CORD HAEMATOMA
97	SKIN ULCER HAEMORRHAGE	288	ORAL MUCOSA HAEMATOMA
98	PERITONEAL HAEMATOMA	289	SPINAL SUBARACHNOID HAEMORRHAGE
99	OESOPHAGEAL ULCER HAEMORRHAGE	290	ARTERIAL INTRAMURAL HAEMATOMA
100	INTRAVENTRICULAR HAEMORRHAGE NEONATAL	291	MUCOCUTANEOUS HAEMORRHAGE
101	RECTAL ULCER HAEMORRHAGE	292	SPINAL SUBDURAL HAEMORRHAGE
102	BLOODY DISCHARGE	293	CHEST WALL HAEMATOMA
103	PEPTIC ULCER HAEMORRHAGE	294	SUBARACHNOID HAEMATOMA
104	OCULAR RETROBULBAR HAEMORRHAGE	295	BONE MARROW HAEMORRHAGE
105	PERIORBITAL HAEMATOMA	296	BURSAL HAEMATOMA
106	PUTAMEN HAEMORRHAGE	297	INTESTINAL VARICES HAEMORRHAGE
107	LIP HAEMORRHAGE	298	JOINT MICROHAEMORRHAGE
108	BRAIN STEM HAEMORRHAGE	299	OESOPHAGEAL INTRAMURAL HAEMATOMA
109	PALPABLE PURPURA	300	PERIOSTEAL HAEMATOMA
110	URINARY OCCULT BLOOD POSITIVE	301	NASAL SEPTUM HAEMATOMA
111	SPLENIC HAEMORRHAGE	302	BASAL GANGLIA HAEMATOMA
112	OCCULT BLOOD POSITIVE	303	CEREBELLAR STROKE
113	OPTIC DISC HAEMORRHAGE	304	TRAUMATIC INTRACRANIAL HAEMATOMA
114	RENAL HAEMATOMA	305	EYE HAEMATOMA
115	SPLENIC HAEMATOMA	306	PARANASAL SINUS HAEMORRHAGE
116	TONGUE HAEMORRHAGE	307	ANAL FISSURE HAEMORRHAGE
117	SCLERAL HAEMORRHAGE	308	SUBGALEAL HAEMORRHAGE
118	CEREBELLAR HAEMATOMA	309	EXTRA-AXIAL HAEMORRHAGE
119	PANCREATIC HAEMORRHAGE	310	PERIVENTRICULAR HAEMORRHAGE NEONATAL
120	MENINGORRHAGIA	311	BLOOD LOSS ANAEMIA
121	TRAUMATIC INTRACRANIAL HAEMORRHAGE	312	HAEMATOMA MUSCLE
122	HAEMATURIA TRAUMATIC	313	PITUITARY APOPLEXY
123	TRAUMATIC HAEMORRHAGE	314	HAEMORRHAGIC ADRENAL INFARCTION

124	INTRACRANIAL HAEMATOMA	315	PULMONARY HAEMORRHAGE NEONATAL
125	HAEMORRHAGIC ASCITES	316	SPINAL STROKE
126	PROCTITIS HAEMORRHAGIC	317	VERTEBROBASILAR STROKE
127	SPINAL CORD HAEMORRHAGE	318	SCROTAL HAEMORRHAGE
128	HAEMATOSALPINX	319	HAEMOPERITONEUM
129	RESPIRATORY TRACT HAEMORRHAGE	320	SUBCAPSULAR RENAL HAEMATOMA
130	TRACHEAL HAEMORRHAGE	321	ORAL BLOOD BLISTER
131	TONGUE HAEMATOMA	322	SUBCAPSULAR HEPATIC HAEMATOMA
132	TRAUMATIC HAEMATOMA	323	BULLOUS HAEMORRHAGIC DERMATOSIS
133	ANAL HAEMORRHAGE	324	ORAL PURPURA
134	VAGINAL HAEMATOMA	325	PANCREATIC PSEUDOCYST HAEMORRHAGE
135	POSTMENOPAUSAL HAEMORRHAGE	326	ORBITAL HAEMATOMA
136	MEDIASTINAL HAEMORRHAGE	327	SUBCAPSULAR SPLENIC HAEMATOMA
137	PENILE HAEMORRHAGE	328	URINARY BLADDER HAEMATOMA
138	SUBCUTANEOUS HAEMATOMA	329	BRONCHIAL VARICES HAEMORRHAGE
139	MEDIASTINAL HAEMATOMA	330	SUBENDOCARDIAL HAEMORRHAGE
140	MYOCARDIAL HAEMORRHAGE	331	FOTHERGILL SIGN POSITIVE
141	EXTRAVASATION BLOOD	332	CEREBRAL CYST HAEMORRHAGE
142	SOFT TISSUE HAEMORRHAGE	333	ORBITAL HAEMORRHAGE
143	STROKE IN EVOLUTION	334	VASCULAR ANASTOMOTIC HAEMORRHAGE
144	LARGE INTESTINAL ULCER HAEMORRHAGE	335	INTRATUMOURAL HAEMATOMA
145	PELVIC HAEMATOMA	336	HEAVY MENSTRUAL BLEEDING
146	INTRACRANIAL TUMOUR HAEMORRHAGE	337	INTERMENSTRUAL BLEEDING
147	URETHRAL HAEMORRHAGE	338	ABNORMAL UTERINE BLEEDING
148	SCROTAL HAEMATOMA	339	URINARY OCCULT BLOOD
149	RETROPLACENTAL HAEMATOMA	340	HAEMORRHAGIC GASTROENTERITIS
150	SPLINTER HAEMORRHAGES	341	JUGULAR VEIN HAEMORRHAGE
151	ADRENAL HAEMORRHAGE	342	HAEMORRHAGIC CEREBELLAR INFARCTION
152	HAEMORRHAGE SUBEPIDERMAL	343	HEPATIC ARTERY HAEMORRHAGE
153	LARGE INTESTINAL HAEMORRHAGE	344	GASTROINTESTINAL ANASTOMOTIC HAEMORRHAGE

154	OPTIC NERVE SHEATH HAEMORRHAGE	345	ABNORMAL MENSTRUAL CLOTS
155	GASTRODUODENAL HAEMORRHAGE	346	THALAMIC STROKE
156	PROSTATIC HAEMORRHAGE	347	OMENTAL HAEMORRHAGE
157	ARTERIOVENOUS FISTULA SITE HAEMORRHAGE	348	SCALP HAEMATOMA
158	HAEMORRHAGE FOETAL	349	SCLERAL HAEMATOMA
159	TESTICULAR HAEMORRHAGE	350	VOCAL CORD HAEMORRHAGE
160	SMALL INTESTINAL ULCER HAEMORRHAGE	351	GALLBLADDER HAEMATOMA
161	CEREBRAL HAEMORRHAGE FOETAL	352	PERIPHERAL VASCULAR HAEMATOMA
162	CORNEAL BLEEDING	353	PARIETAL LOBE STROKE
163	OSTEORRHAGIA	354	MIDDLE CEREBRAL ARTERY STROKE
164	PARATHYROID HAEMORRHAGE	355	OCCIPITAL LOBE STROKE
165	PAPILLARY MUSCLE HAEMORRHAGE	356	GASTROOESOPHAGEAL HAEMORRHAGE
166	NIPPLE EXUDATE BLOODY	357	INTRACRANIAL HAEMORRHAGE NEONATAL
167	POST ABORTION HAEMORRHAGE	358	SUBCORTICAL STROKE
168	NAIL BED BLEEDING	359	SPINAL HAEMATOMA
169	CHOROIDAL HAEMORRHAGE	360	FAECAL OCCULT BLOOD POSITIVE
170	HAEMOBILIA	361	MENORRHAGIA
171	COLONIC HAEMATOMA	362	PERITONEAL HAEMORRHAGE
172	ANASTOMOTIC ULCER HAEMORRHAGE	363	SKIN BLEEDING
173	IRIS HAEMORRHAGE	364	PERIRENAL HAEMATOMA
174	SUBARACHNOID HAEMORRHAGE NEONATAL	365	ANTEPARTUM HAEMORRHAGE
175	HYPHAEMA	366	COLONIC HAEMORRHAGE
176	THORACIC HAEMORRHAGE	367	ABDOMINAL HAEMATOMA
177	CHRONIC GASTROINTESTINAL BLEEDING	368	CEREBRAL HAEMORRHAGE TRAUMATIC
178	PURPURA SENILE	369	PLEURAL HAEMORRHAGE
179	OVARIAN HAEMATOMA	370	POST COITAL BLEEDING
180	EYELID BLEEDING	371	DYSFUNCTIONAL UTERINE BLEEDING
181	PULMONARY HAEMATOMA	372	ORAL MUCOSAL PETECHIAE
182	HAEMORRHAGE CORONARY ARTERY	373	BLEEDING PERIPARTUM
183	CERVIX HAEMORRHAGE UTERINE	374	SCHAMBERG'S DISEASE
184	HAEMORRHAGIC CYST	375	HAEMATOMETRA
185	TONSILLAR HAEMORRHAGE	376	GASTRODUODENITIS HAEMORRHAGIC

186	VULVAL HAEMORRHAGE	377	BLOODY AIRWAY DISCHARGE
187	PLACENTA PRAEVIA HAEMORRHAGE	378	MAJOCCHI'S PURPURA
188	SUBDURAL HAEMORRHAGE NEONATAL	379	HAEMORRHAGIC ANAEMIA
189	UROGENITAL HAEMORRHAGE	380	HAEMATOMYELIA
190	HAEMORRHAGIC INFARCTION	381	INTESTINAL POLYP HAEMORRHAGE
191	SKIN HAEMORRHAGE	382	PERIPHERAL ARTERY HAEMATOMA

Table S2 The results of logistic regression analysis.

	Variable	OR	CI_lower	CI_upper	P_value	95% CI
DE&Levo	AGE	1.01	1.01	1.01	3.58E-53	1.01（1.01-1.01）
	WT	1.00	1.00	1.00	8.71E-06	1（1-1）
	SEX	1.15	1.11	1.20	2.25E-11	1.15（1.11-1.2）
	Indi_AF	1.84	1.75	1.92	8.83E-143	1.84（1.75-1.92）
	Levo combination	6.12	3.66	11.01	8.74E-11	6.12（3.66-11.01）
	DE	1.54	1.47	1.62	8.88E-68	1.54（1.47-1.62）
DE&Moxi	AGE	1.01	1.01	1.01	1.37E-53	1.01（1.01-1.01）
	WT	1	1	1	6.15E-06	1（1-1）
	SEX	1.15	1.1	1.2	4.18E-11	1.15（1.1-1.2）
	Indi_AF	1.86	1.77	1.95	1.51E-148	1.86（1.77-1.95）
	Moxi combination	2.37	0.76	8.86	0.154964636	2.37（0.76-8.86）
	DE	1.52	1.45	1.6	1.11E-64	1.52（1.45-1.6）
DE&Cipro	AGE	1.01	1.01	1.01	2.48E-53	1.01（1.01-1.01）
	WT	1	1	1	7.75E-06	1（1-1）
	SEX	1.15	1.11	1.2	2.31E-11	1.15（1.11-1.2）
	Indi_AF	1.84	1.76	1.93	9.06E-144	1.84（1.76-1.93）
	Cipro combination	3.84	2.52	6.06	1.54E-09	3.84（2.52-6.06）
	DE	1.54	1.46	1.61	2.50E-67	1.54（1.46-1.61）
Riva&Levo	AGE	1.01	1.01	1.02	1.04E-62	1.01（1.01-1.02）
	WT	1	1	1	1.42E-07	1（1-1）
	SEX	1.15	1.1	1.2	7.94E-11	1.15（1.1-1.2）

	Indi_AF	2.36	2.26	2.46	0	2.36（2.26-2.46）
	Levo combination	0.34	0.21	0.52	2.87E-06	0.34（0.21-0.52）
	Riva	1.23	1.17	1.28	2.18E-19	1.23（1.17-1.28）
	AGE	1.01	1.01	1.02	2.88E-63	1.01（1.01-1.02）
	WT	1	1	1	1.37E-07	1（1-1）
	SEX	1.15	1.1	1.2	1.15E-10	1.15（1.1-1.2）
Riva&Moxi	Indi_AF	2.37	2.27	2.48	0	2.37（2.27-2.48）
	Moxi combination	0.92	0.32	2.43	0.870375075	0.92（0.32-2.43）
	Riva	1.23	1.18	1.29	1.53E-20	1.23（1.18-1.29）
	AGE	1.01	1.01	1.02	2.99E-63	1.01（1.01-1.02）
	WT	1	1	1	1.44E-07	1（1-1）
	SEX	1.15	1.1	1.2	1.16E-10	1.15（1.1-1.2）
Riva&Cipro	Indi_AF	2.37	2.27	2.47	0	2.37（2.27-2.47）
	Cipro combination	0.86	0.56	1.28	0.46073915	0.86（0.56-1.28）
	Riva	1.23	1.18	1.29	2.28E-20	1.23（1.18-1.29）
	AGE	1.02	1.01	1.02	3.57E-72	1.02（1.01-1.02）
	WT	1	1	1	9.44E-08	1（1-1）
	SEX	1.15	1.1	1.2	4.98E-11	1.15（1.1-1.2）
Api&Levo	Indi_AF	2.02	1.94	2.11	1.40E-231	2.02（1.94-2.11）
	Levo combination	0.46	0.32	0.67	5.63E-05	0.46（0.32-0.67）
	Api	0.59	0.57	0.62	7.11E-107	0.59（0.57-0.62）
Api&Moxi	AGE	1.02	1.01	1.02	2.17E-71	1.02（1.01-1.02）

	WT	1	1	1	8.84E-08	1（1-1）
	SEX	1.15	1.1	1.2	4.94E-11	1.15（1.1-1.2）
	Indi_AF	2.03	1.95	2.12	2.66E-234	2.03（1.95-2.12）
	Moxi combination	0	#VALUE!	0.06	0.832639414	#VALUE!
Api&Cipro	Api	0.6	0.57	0.62	1.60E-105	0.6（0.57-0.62）
	AGE	1.02	1.01	1.02	1.79E-72	1.02（1.01-1.02）
	WT	1	1	1	7.84E-08	1（1-1）
	SEX	1.15	1.1	1.2	9.97E-11	1.15（1.1-1.2）
	Indi_AF	2.02	1.93	2.11	1.33E-229	2.02（1.93-2.11）
	Cipro combination	0.2	0.12	0.32	3.85E-10	0.2（0.12-0.32）
Edo&Levo	Api	0.59	0.57	0.62	6.08E-108	0.59（0.57-0.62）
	AGE	1.01	1.01	1.01	1.05E-56	1.01（1.01-1.01）
	WT	1	1	1	3.31E-06	1（1-1）
	SEX	1.15	1.1	1.2	3.63E-11	1.15（1.1-1.2）
	Indi_AF	2.25	2.16	2.35	0	2.25（2.16-2.35）
	Levo combination	0.75	0.09	6.29	0.770473911	0.75（0.09-6.29）
Edo&Moxi	Edo	0.64	0.56	0.74	4.42E-10	0.64（0.56-0.74）
	AGE	1.01	1.01	1.01	1.04E-56	1.01（1.01-1.01）
	WT	1	1	1	3.36E-06	1（1-1）
	SEX	1.15	1.1	1.2	3.91E-11	1.15（1.1-1.2）
	Indi_AF	2.25	2.16	2.35	0	2.25（2.16-2.35）
	Moxi combination	0.34	0.02	2.35	0.336807258	0.34（0.02-2.35）

	Edo	0.64	0.56	0.74	4.39E-10	0.64（0.56-0.74）
Edo&Cipro	AGE	1.01	1.01	1.01	4.24E-57	1.01（1.01-1.01）
	WT	1	1	1	3.42E-06	1（1-1）
	SEX	1.15	1.11	1.2	2.46E-11	1.15（1.11-1.2）
	Indi_AF	2.26	2.16	2.35	0	2.26（2.16-2.35）
	Cipro combination	0	——	0	0.862827503	——
	Edo	0.64	0.56	0.74	3.90E-10	0.64（0.56-0.74）

Api apixaban, *Cipro* ciprofloxacin*,* *DE* dabigatran etexilate, *Edo* edoxaban, *FQNs* fluoroquinolones, *Indi_AF* indications for atrial fibrillation, *Levo* levofloxacin, *Moxi* moxifloxacin, *Riva* rivaroxaban

WT weight

Table S3. Demographic data and concomitant medication in studied patients with atrial fibrillation on dabigatran therapy.

	DE	DE + FQNs	DE + Moxi	DE + Levo	*F* (DE + FQNs) vs	*P* value (DE + FQNs) vs
Demographic						
Number of patients	30	20	12	8	N/A	N/A
Male，n(%)	21（70%）	8（40%）	4（33.33%）	4（50%）	6.704	0.073
Age, year	73.60 ± 7.16	77.15 ± 8.48	76.83 ± 8.79	77.63 ± 8.57	1.143	0.338
Body weight, kg	70.98 ± 12.45	64.65 ± 15.11	62.42 ± 14.51	68.00 ± 16.35	1.415	0.246
BMI, kg/m2	25.84 ± 4.12	24.30 ± 3.93	23.72 ± 3.61	25.16 ± 4.47	1.044	0.379
CrCl, mL/min	70.06 ± 19.47	64.19 ± 22.77	65.96 ± 25.40	61.54 ± 19.48	0.491	0.690
AST, U/L	19.31 ± 6.05	21.25 ± 7.01	20.00 ± 7.21	23.13 ± 6.71	0.839	0.478
ALT, U/L	18.48 ± 13.30	22.45 ± 20.22	22.33 ± 17.88	22.63 ± 24.63	0.280	0.839
Blood pressure, mmHg						
SBP	129.77 ± 20.31	137.95 ± 21.87	140.33 ± 19.07	134.38 ± 26.50	0.968	0.413
DBP	77.83 ± 18.10	82.25 ± 18.10	83.25 ± 13.88	80.75 ± 24.14	0.366	0.778
Risk of thrombosis and						
CHA2DS2-VASc	3.13 ± 1.46	4.05 ± 1.64	4.42 ± 1.73	3.50 ± 1.41	2.546	0.063
HAS-BLED	0.90 ± 0.80	1.20 ± 0.62	1.25 ± 0.62	1.13 ± 0.64	1.078	0.365
Medical history, n (%)						
Hypertension	23（76.67%）	15（75%）	10（83.33%）	5（62.50%）	1.279	0.731
Stroke	7（23.33%）	2（10%）	2（16.67%）	0	2.718	0.441
Diabetes mellitus	6（20%）	6（30%）	4（33.33%）	2（25%）	1.313	0.769

ACE angiotensin-converting enzyme, *AST* Aspartate Aminotransferase, *ALT* Alanine Aminotransferase, *AT1RB* AT1 receptor blockers, *BMI* body mass index, *CrC* Creatinine Clearance, *DE* dabigatran etexilate, *FQNs* fluoroquinolones, *Levo* levofloxacin, *Moxi* moxifloxacin
